# Dataset of operating conditions to Isolate Cellulose Nanocrystalline from Sugarcane Bagasse and Pinewood Sawdust as Possible Material to Fabricate Polymer Electrolyte Membranes

**DOI:** 10.1016/j.dib.2020.105597

**Published:** 2020-04-23

**Authors:** A. Macías-Almazán, J.A. Lois-Correa, M.A. Domínguez-Crespo, A.B. López-Oyama, A.M. Torres-Huerta, S.B. Brachetti-Sibaja, A.E. Rodríguez-Salazar

**Affiliations:** a Instituto Politécnico Nacional, Centro de Investigación en Ciencia Aplicada y Tecnología Avanzada CICATA, Unidad Altamira, Carretera Tampico-Puerto Industrial, km 14.5, Altamira, Tamaulipas, CP 89600, México; b Instituto Politécnico Nacional UPII Hidalgo, Ciudad del Conocimiento y la Cultura, Carretera Pachuca - Actopan km 1+500, San Agustín Tlaxiaca, C.P. 42162, Hgo, México; c CONACYT – CICATA- Altamira, Carretera Tampico-Puerto Industrial Altamira, C. P. 89600. Altamira, Tamps, México; d TecNM, Instituto Tecnológico de Cd. Madero, Ave. Primero de Mayo s/n Col. Los Mangos Cd. Madero Tamps, C.P. 89440; e Instituto Politécnico Nacional, Centro de Investigación en Ciencia Aplicada y Tecnología Avanzada Unidad-Querétaro, Cerro Blanco No. 141 Col. Colinas del Cimatario, C.P. 76090, Querétaro, Qro, México

**Keywords:** Agroindustrial Wastes, Sugarcane Bagasse, Pinewood Sawdust, Nanocellulose, PEMFCs

## Abstract

The data shown in this document provides all the experimental data that complement the article published in Carbohydrate Polymers entitled “Influence of operating conditions on Proton Conductivity of Nanocellulose films using two Agroindustrial Wastes: Sugarcane Bagasse and Pinewood Sawdust” [1]. The data of this paper are the result of a large series of experiments to optimize the extraction of cellulose nanocrystalline (CNC) from these two agro-industrial wastes: sugarcane Bagasse (SCB) and pinewood sawdust (PSW). The conditions of pretreatment (5 wt.% or 10 wt.% of NaOH) and hydrolysis temperature (60, 75 and 90°C) in an aqueous solution of 45 wt.% of H_2_SO_4_ were analyzed exhaustively. The data includes the characterization by Fourier transform infrared (FT-IR), Differential Scanning Calorimetry/Thermogravimetric Analysis (DSC/TGA), Dynamic Light Scattering (DLS), X-ray diffraction (XRD) patterns, Scanning Electron Microscopy (SEM), Transmission Electron Microscopy (TEM) micrographs with their corresponding SAED patterns and nanoindentation tests. Additionally, photographs during the isolation of cellulose nanocrystalline in dependence of the syntheses parameters. It is also included the data that complement the molecular dynamic simulation generated by GLYCAM carbohydrate builder based on the coordinates for alpha and beta cellulose considering a microfibril of 5, 10 and 20 glucosyl residues (degree of polymerization, DP). Overall data have not been previously published and are available contributing to a better understanding of the CNCs isolation through different pretreatment concentrations and temperatures of processing.

Specifications table

**Table:** 

Subject	Materials science
Specific subject area	Nanotechnology
Type of data	Table Image Figure Photographs Schema
How data were acquired	Samples were prepared for characterization as described in [1]. The characterization was realized by X-ray diffraction (XRD, Brucker D8 Advanced), FTIR (Perkin Elmer Spectrun One FTIR spectrometer), Dynamic Light Scattering (DLS) using a Litesizer 500 equipment (40 mW semiconductor laser of 658 nm wavelength; Particle Analyzer, Anton Paar.) Scanning Electron Microscopy (JEOL JSM-6701F microscope), Transmission electron microscopy (JEOL JEM-2000 FX). MD simulations were produced with AmberTools suite. The mechanical properties were carried out an Anton Paar Nanoindentation Tester NHT^3^. DSC/TGA was analyzed in the SETARAM Instrumentation Labsys Evo equipment.
Data format	Raw. Mendeley Data http://dx.doi.org/10.17632/z5cz9n78j7.2. Analyzed Filtered Fitted curves
Parameters for data collection	The parameters for data collection were the concentration of NaOH (5 and 10 wt. %), temperature of acid hydrolysis (45 wt. % H_2_SO_4_, 60, 75 and 90°C) and for MD the number of glucosyl residues (5, 10 and 20).
Description of data collection	The data were collected after the isolation of cellulose nanocrystals from two-agroindustrial wastes: sugarcane bagasse and pinewood sawdust powders, varying the concentration of NaOH pretreatment and hydrolysis temperature (H_2_SO_4_).
Data source location	SEM and TEM images were taken at ESIQIE of the Instituto Politécnico Nacional, C.P. 07738 Ciudad de México, CDMX, México. FT-IR and DLS techniques, mechanical properties, XRD patterns and Analysis DSC/TGA analysis were collected at Centro de investigación en Ciencia Aplicada y Tecnología Avanzada del Instituto Politécnico Nacional, C.P. 89600 Altamira, Tamaulipas, México.
Data accessibility	All data are available with this article and in Medeley Data. http://dx.doi.org/10.17632/z5cz9n78j7.2
Related research article	Macías-Almazán, J. A. Lois-Correa, M. A. Domínguez-Crespo, A. B. López-Oyama, A. M. Torres-Huerta, S. B. Brachetti-Sibaja, A. E. Rodríguez-Salazar. Influence of operating conditions on Proton Conductivity of Nanocellulose films using two Agroindustrial Wastes: Sugarcane Bagasse and Pinewood Sawdust. Carbohydrate Polymers. 238(2020)116171 https://doi.org/10.1016/j.carbpol.2020.116171.

## Value of the data

• The data are useful since show the importance of adjust the NaOH pretreatment concentration and temperature of acid hydrolysis (H_2_SO_4_) to isolate cellulose nanocrystalline.
• The data help to understand the connection between the crystallinity, proton conductivity and mechanical properties.
• The agroindustrial sector can be helped to process, the by-products into products of high added value, for example during the fabrication of polymer electrolyte membranes.
• The data show the MD simulation from which the researchers can evaluate the behavior of the donor-acceptor of CNCs under the conditions of this research.

## 1 Data description

**Fig. 1:**
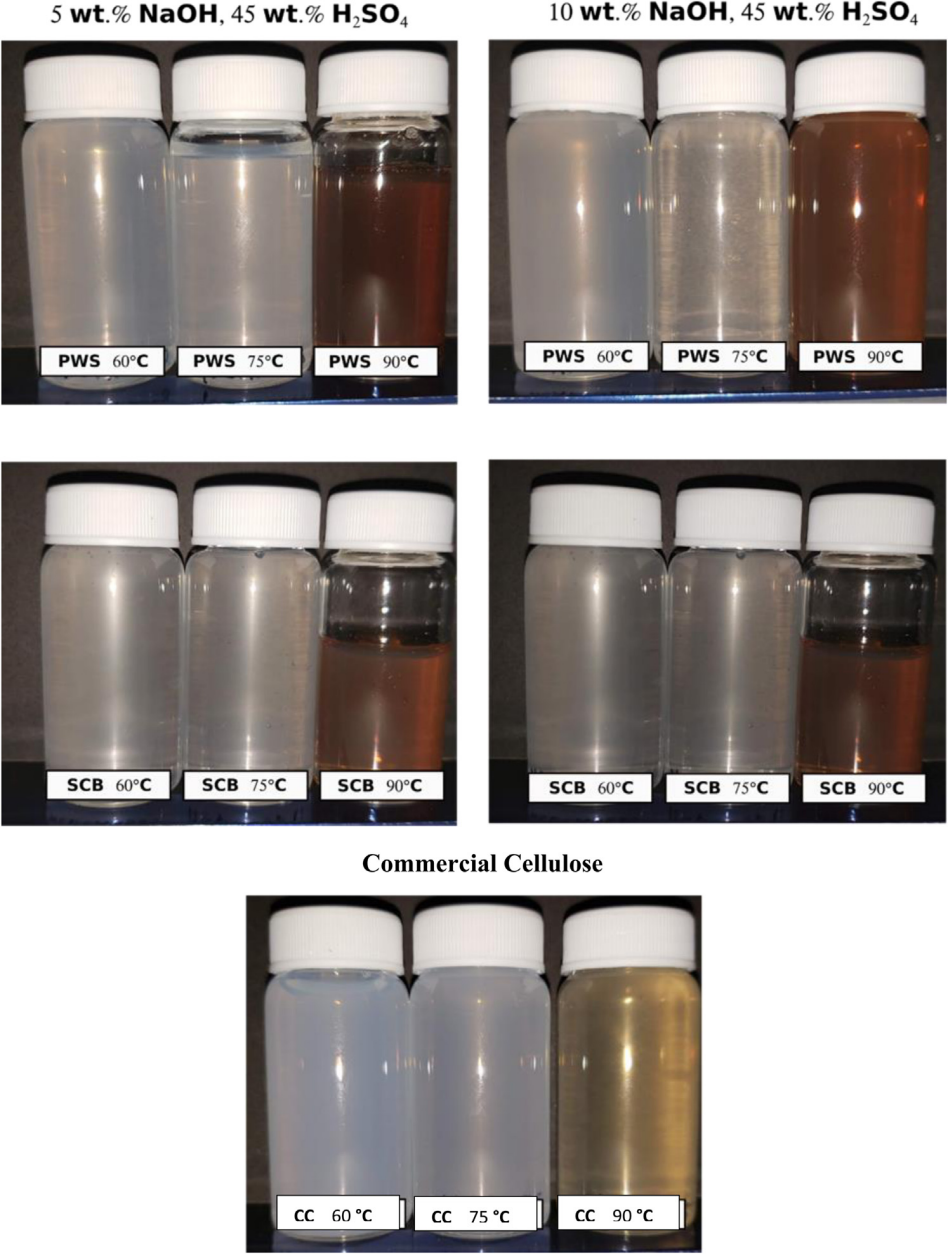
Photographic record of the nanocrystalline cellulose produced from agroindustrial wastes with 5 and 10 wt.%. NaOH, 45 wt.%. H_2_SO_4_ under different temperatures.

**Fig. 2:**
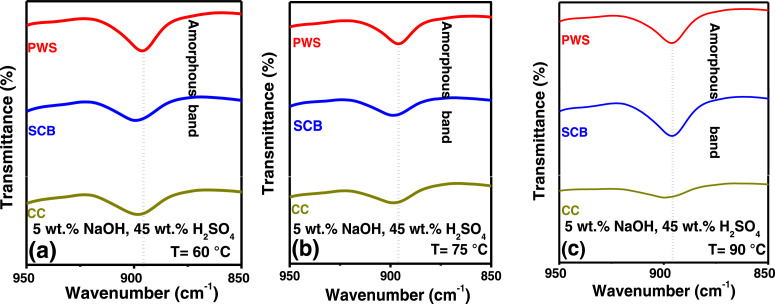
FTIR spectra of crystalline nanocellulose from CC, SCB and PWS produced with 5 wt.% NaOH, 45 wt.% H_2_SO_4_ at (a) 60°C, (b) 75°C and (c) 90°C.

**Fig. 3:**
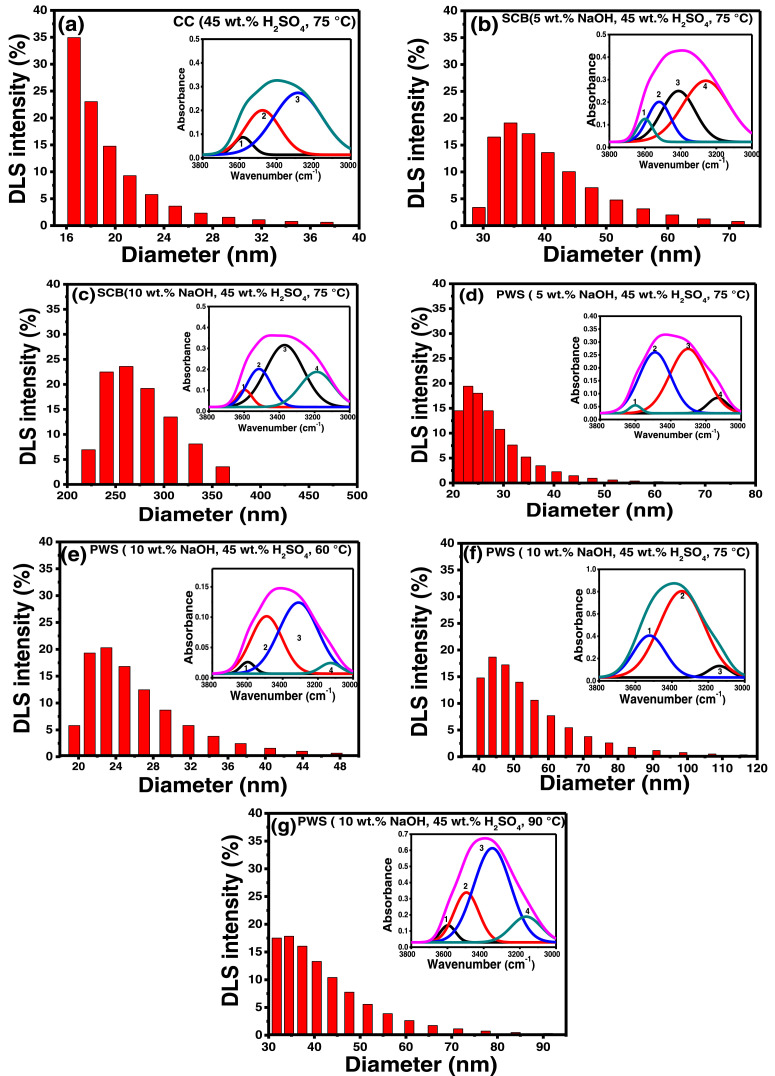
Particle size distribution histograms of crystalline nanocellulose obtained from DLS measurements. The inset figures shown deconvolution of FT-IR spectra in the 3800-3000 cm^−1^ region for all the samples to observe inner components of hydrogen-bonded OH stretching.

**Fig. 4:**
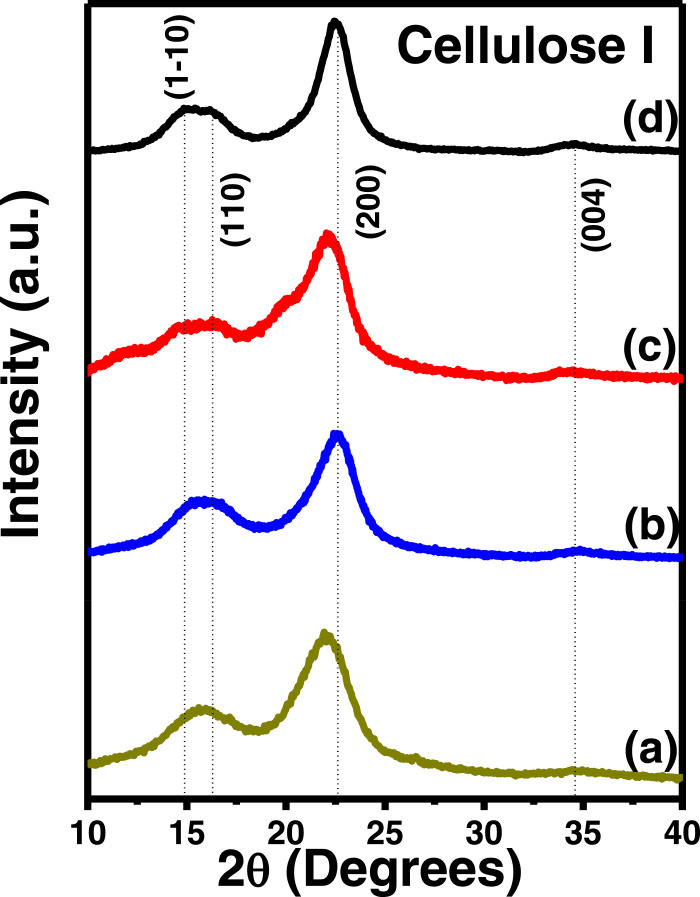
XRD patterns of (a) raw SCB, (b) SCB cellulose produced with 5 wt.% NaOH, 45 wt.% H_2_SO_4_, (c) SCB cellulose produced with 10 wt.% NaOH, 45 wt.% H_2_SO_4_ and (d) Commercial cellulose using 75°C.

**Fig. 5:**
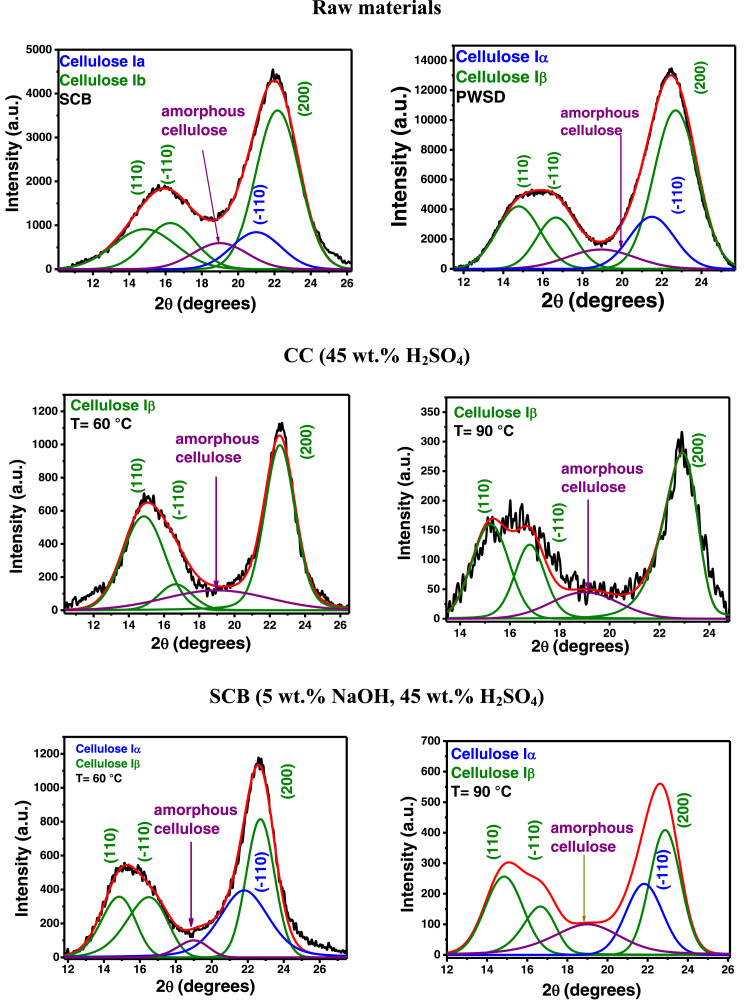
Separation of amorphous and crystalline phases in dependence of synthesis parameters, using the Shirley background subtraction with a Gaussian- Lorentzian profile, of raw materials, CC (45 wt.% H_2_SO_4_) and SCB (5 wt.% NaOH, 5 wt.% H_2_SO_4_).

**Fig. 6:**
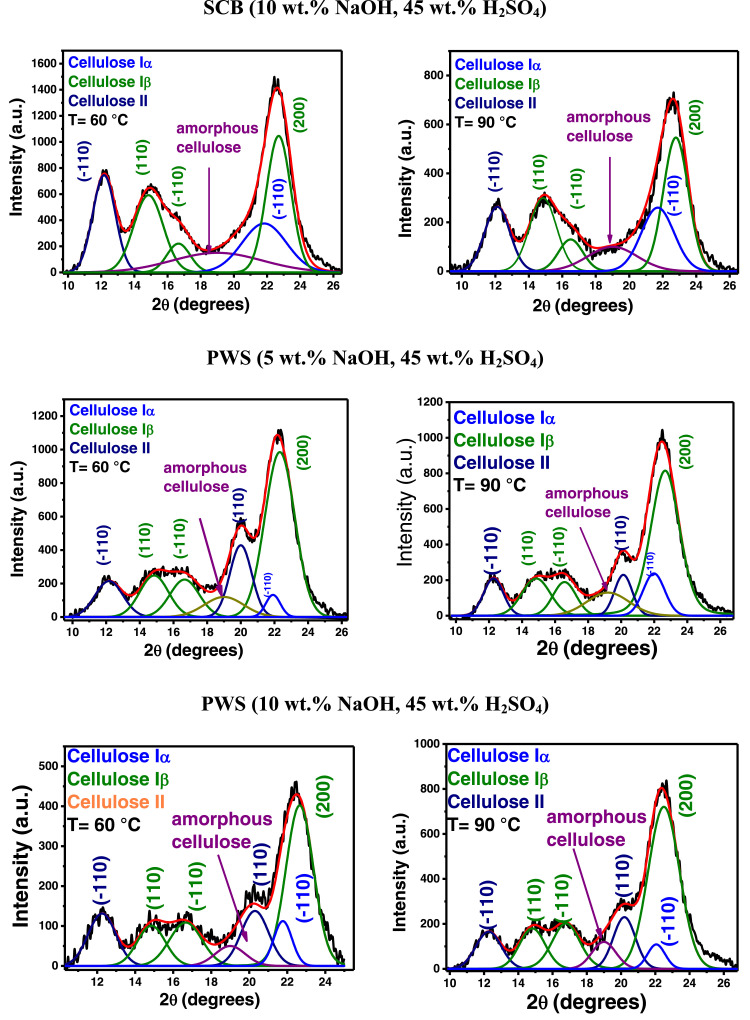
Separation of amorphous and crystalline phases in dependence of synthesis parameters, using the Shirley background subtraction with a Gaussian- Lorentzian profile of SCB (10 wt.% NaOH, 5 wt.% H_2_SO_4_, PWS (5 wt.% NaOH, 5 wt.% H_2_SO_4_ and PWS (10 wt.% NaOH, 5 wt.% H_2_SO_4_).

**Fig. 7:**
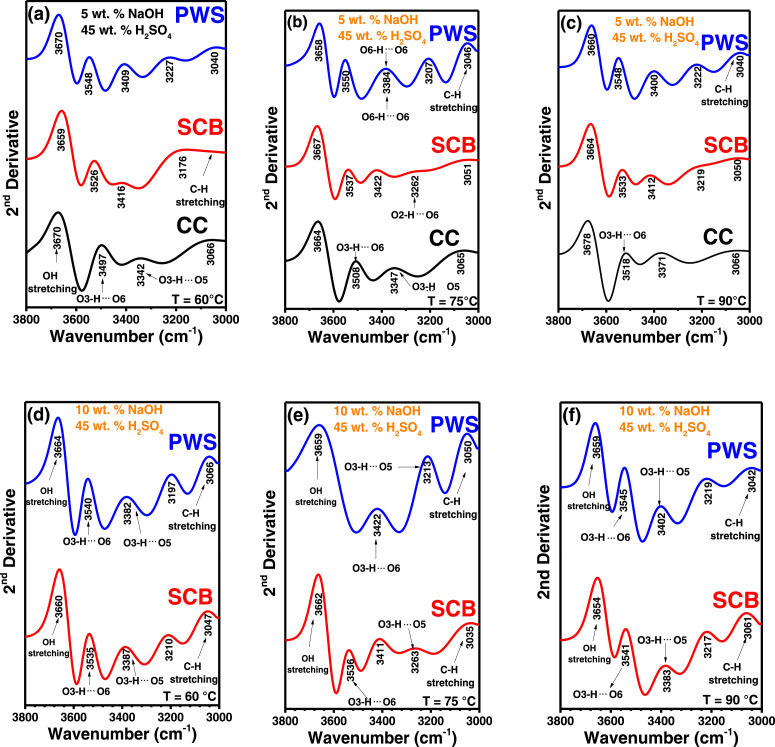
2^nd^ derivative of FTIR region 3800-3000 cm^−1^ of H-bonds that compose the -OH stretching band of the nanocellulose by varying the solvent medium and temperature.

**Fig. 8:**
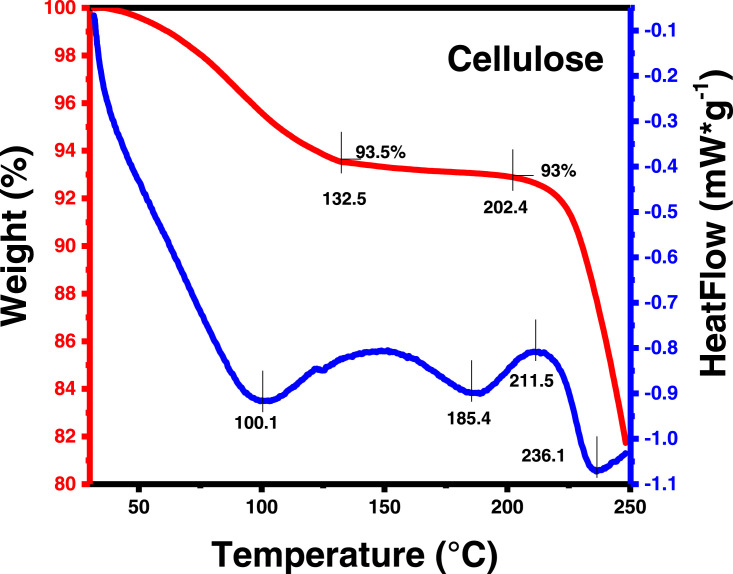
Differential Scanning Calorimetry/Thermogravimetry analysis of SCB at 75°C.

**Fig. 9:**
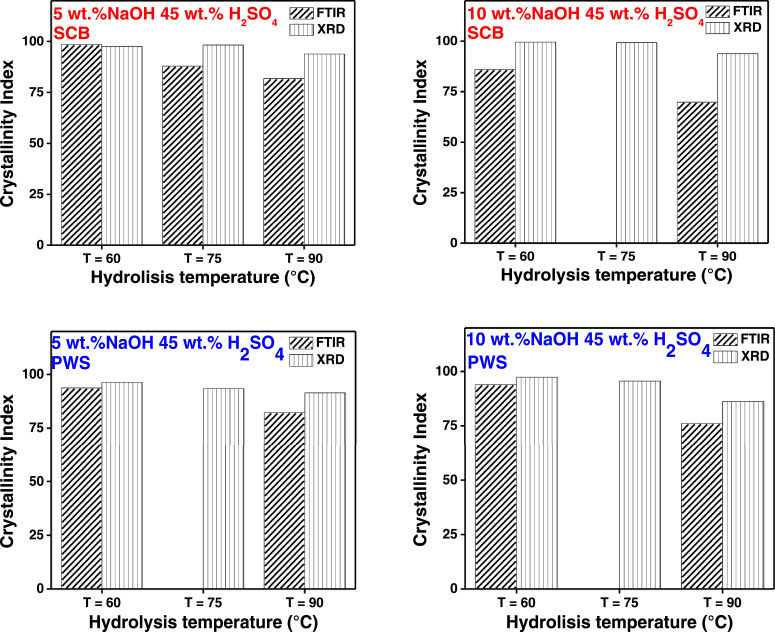
Cellulose crystallinity index comparison of PWS and SCB at 60, 75 and 90°C under different pretreatment conditions for the data acquired from FTIR measurements (sparse pattern) and XRD measurements (dense pattern).

**Fig. 10:**
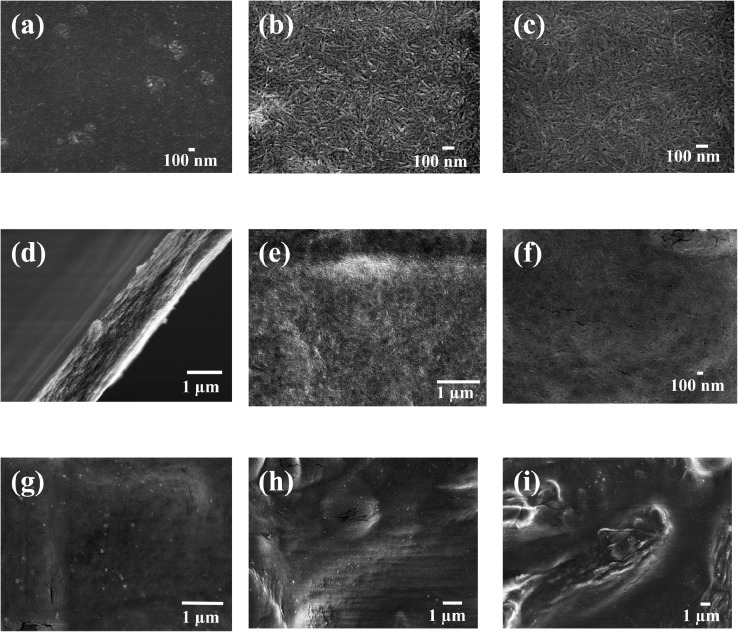
SEM micrographs of isolated nanocrystalline cellulose from CC at different temperatures where it is seen the nanocrystalline and microcrystalline regions. SEM images corresponds to CC at (a-d) 60°C, (e-f) 75°C, (g-i) 90°C.

**Fig. 11:**
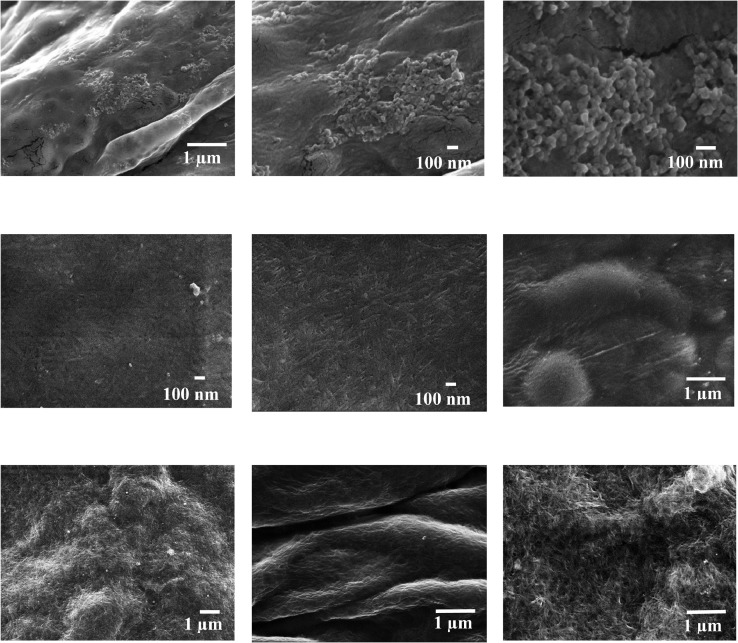
SEM micrographs of isolated nanocrystalline cellulose from PWS and SCB at different temperatures where it is seen the nanocrystalline and microcrystalline regions. SEM images corresponds to PWS at (a-c) 75°C and SCB at (d-f) 60°C, (g-i) 75°C.

**Fig. 12:**
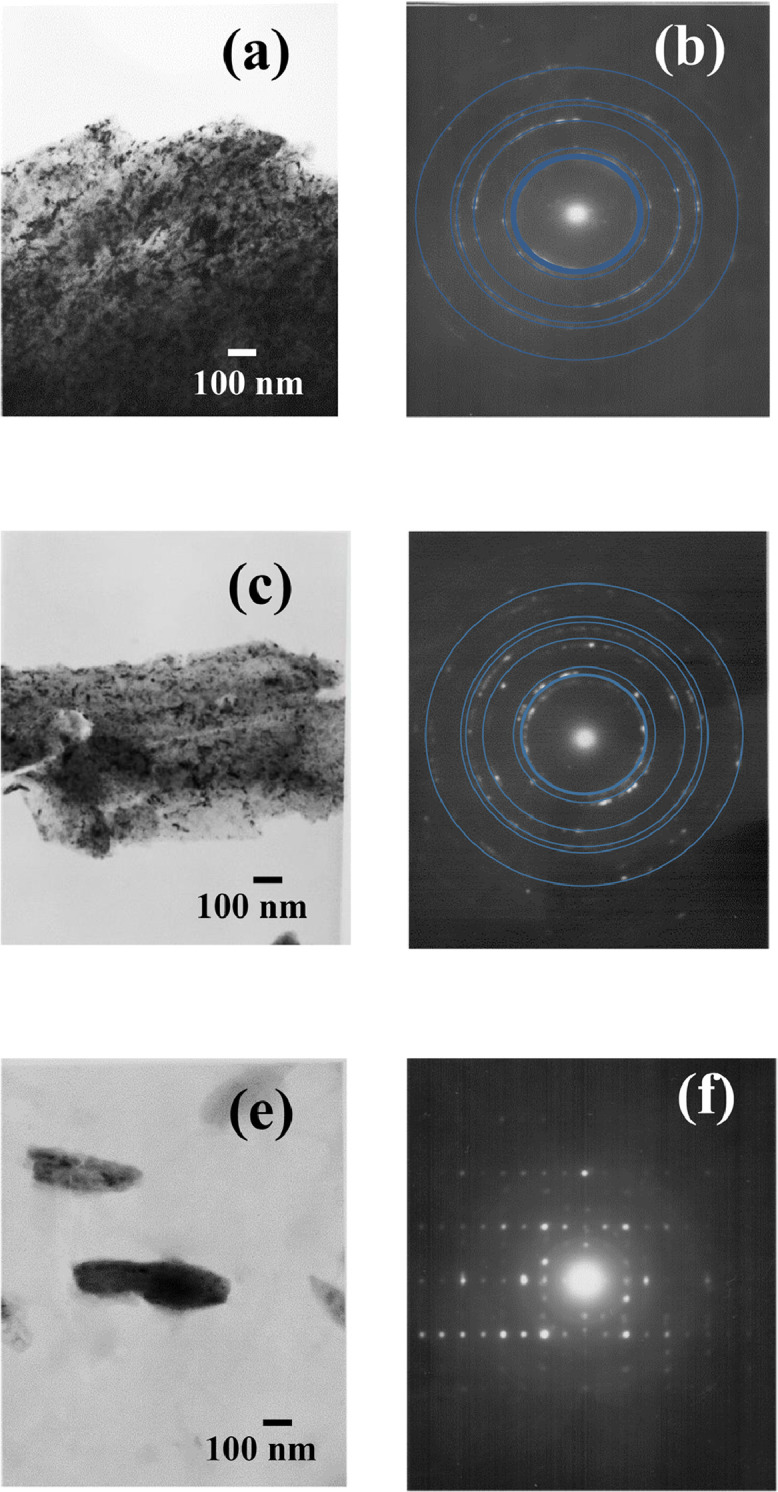
TEM images with its corresponding SAED patterns and isolated nanocrystalline cellulose from CC and SCB at different temperatures where it is seen the nanocrystalline and microcrystalline regions. TEM micrographs are shown for CC at (a-d) 75°C and SCB at (e-f) 75°C. All the samples were prepared with 5 wt.% NaOH followed of an acid hydrolysis with 45 wt.% H_2_SO_4._

**Fig. 13:**
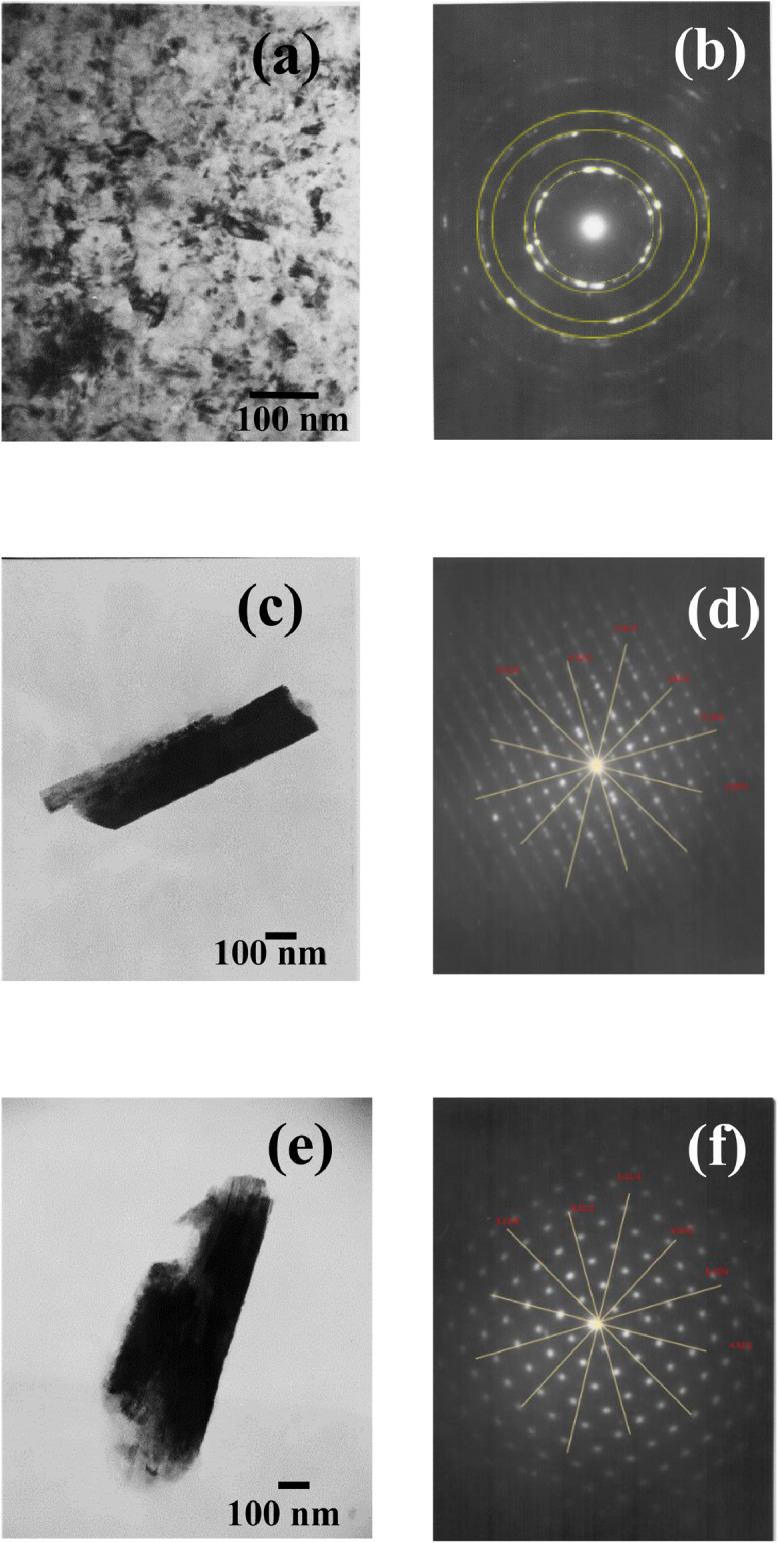
TEM images with its corresponding SAED patterns of isolated nanocrystalline cellulose from PWS and CC at different temperatures where it is seen the nanocrystalline and microcrystalline regions. TEM micrographs are shown for PWS at (a-b) 75°C and CC at (c-f) 60°C.

**Fig. 14:**
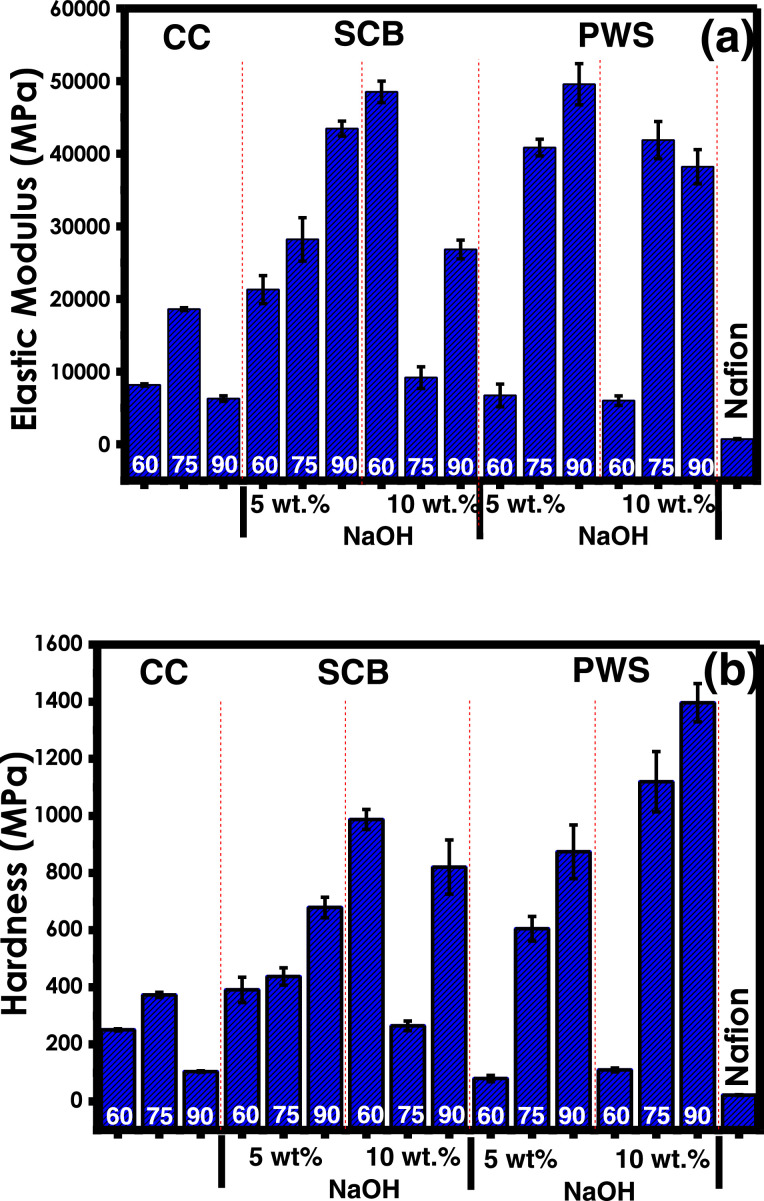
Results of mechanical properties to different parameters such as temperature of synthesis at 60, 75 and 90°C, acid hydrolysis at 45 wt.% H_2_SO_4_ and 5, 10 wt.% of NaOH (a) elastic modulus and (b) hardness at different synthesis conditions.

**Fig. 15:**
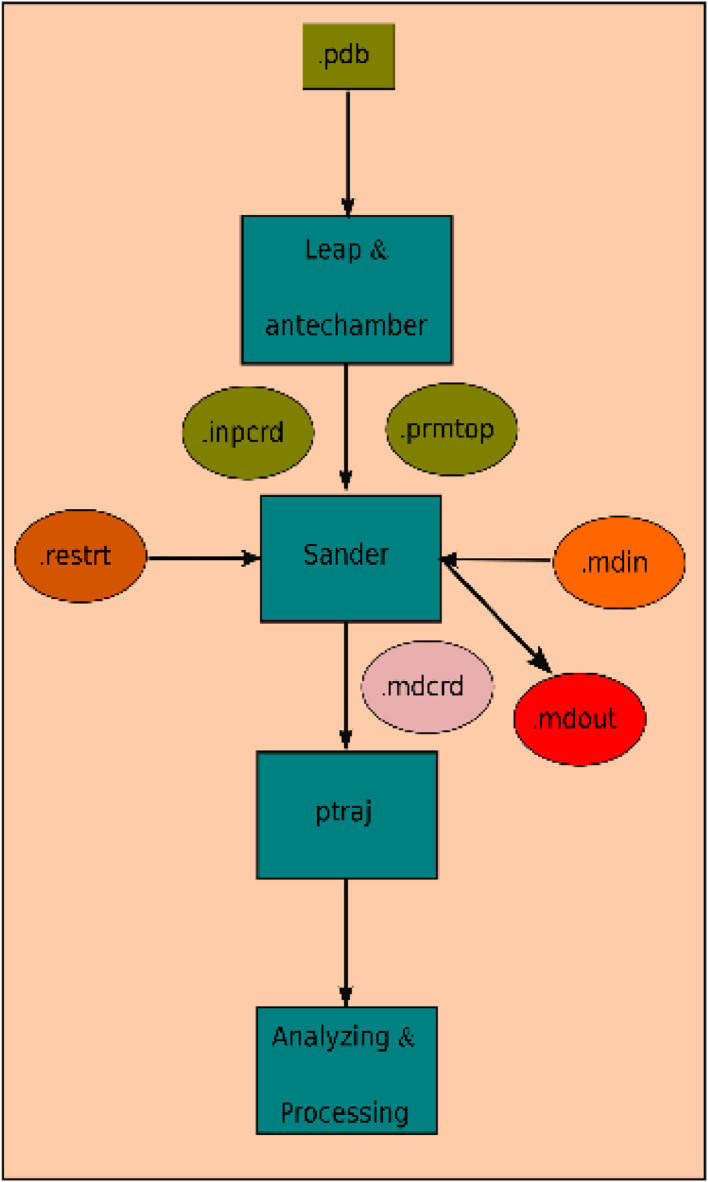
Data analysis flowchart for the initial coordinates to analyze the data for DM simulation by using AmberTools.

**Fig. 16:**
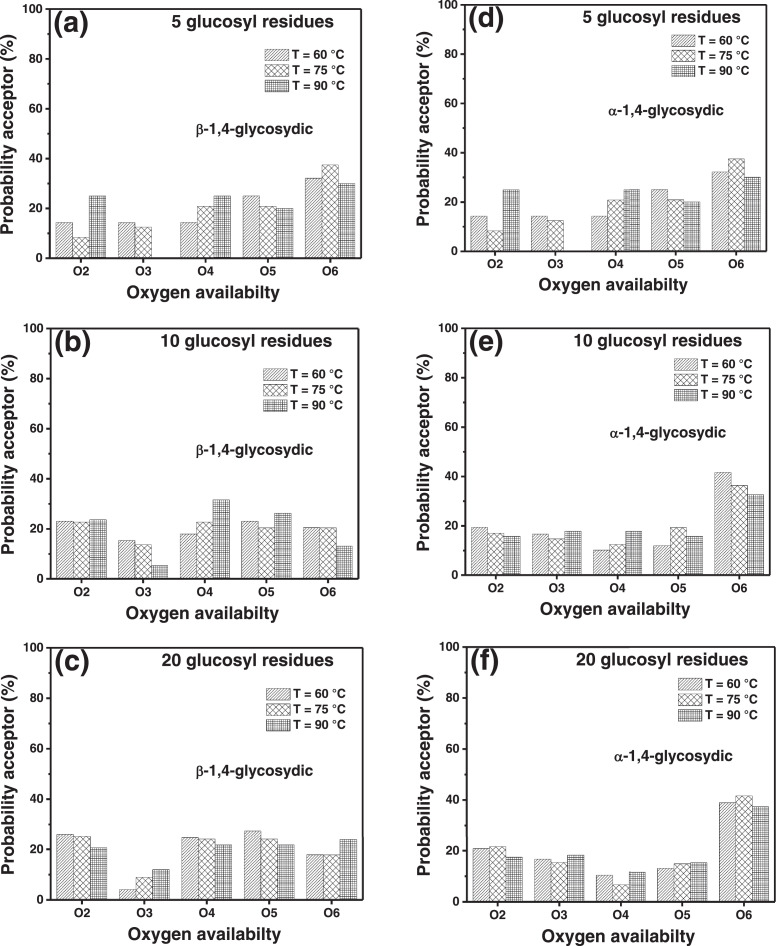
Donor-acceptor probabilities for H-bond formation with the oxygen functionalities into the structures with 5 (a, d), 10 (b, e) and 20 (c, f) residues chain-length of both α-1,4 and β-1,4 linkages of cellulose.

**Fig. 17:**
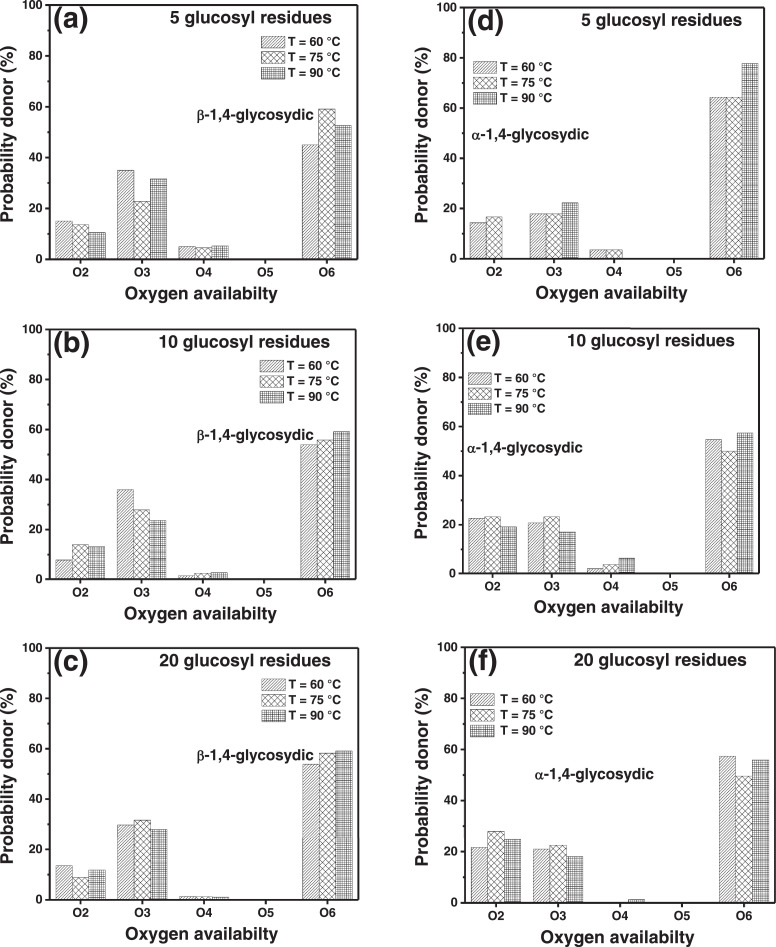
Donor-acceptor probabilities for H-bond formation with the oxygen functionalities into the structures with 5 (a, d), 10 (b, e) and 20 (c, f) residues chain-length of both α-1,4 and β-1,4 linkages of cellulose.

In the present article, we report the data related to the nanocellulose production from two agroindustrial waste materials: SCB and PWS. Fig. 1 shows photographs of isolate cellulose nanocrystalline at each experimental conditions of isolation. The photographs show changes in the appearance in dependence of processing parameters. The dataset includes FT-IR spectra that were plotted from the 950 to 850 cm^−1^ (wavenumber) vs the transmittance (%) that correspond to different treatments applied to SCB and PWS (Fig. 2 a-c). The spectra were shown for each temperature of acid hydrolysis (5 wt. % of NaOH and 45 wt.% of H_2_SO_4_) at 60°C (Fig. 2a), 75°C (Fig. 2b) and 90°C (Fig. 2c). For comparison, it is presented the FT-IR spectra of commercial cellulose (CC). The Fig. 3 displays particle size distribution histograms of crystalline nanocellulose obtained from DLS measurements. The inset figures also showed deconvolution of FT-IR spectra in the 3800-3000 cm^−1^ region for all the samples where it can be observed the inner components of hydrogen-bonded OH stretching. Fig. 4 depicts the XRD patterns scanned from 10-40° in θ-2θ arrangement that includes the results of the SCB raw (Fig. 4a), CNC isolated from SCB with 5 wt.% (Fig. 4b) and 10 wt.% (Fig. 4c) of NaOH followed of the acid hydrolysis with 45 wt.% of H_2_SO_4_ at 75°C, respectively. The structural comparison was realized with commercial cellulose (Fig. 4d). The quantification of amorphous and crystalline phases in dependence of synthesis parameters was carried out by the Shirley background substation with a Gaussian-Lorentzian profile. The Fig. 5, Fig. 6 show the deconvolution of the as-received raw materials, commercial cellulose and the CNCs isolated from each agroindustrial waste. To verify chemical changes in FT-IR bands related with the H-bonds that compose the -OH stretching bands, in the region from 3800 to 3000 cm^−1^, it was realized an analysis using a second derivative (Fig. 7 a-f). This figure shows the second derivative as a function of the evaluate parameters. The DSC/TGA analysis for a typical sample of cellulose nanocrystalline extracted from SCB at 75°C is shown in Fig. 8. A comparison of the crystallinity computed from FT-IR and XRD measurements was shown in Fig. 9. In Fig. 10, Fig. 11, it is presented the morphology of crystalline films on slide glass using a wet film of 120 µm. The microstructural aspects of this films were analyzed by TEM images with their corresponding selected area of electron diffraction (SAED) patterns (Fig. 12, Fig. 13). The mechanical properties of cellulose membranes were performed with a nanoindentation Tester NHT^3^ using a Poisson's ratio of 0.38 [2]. Overall samples were measured 5 times on different points with a loading and unloading rate of 8 mN-min^−1^ with a maximum load of 40 mN which was hold 10 seconds and then unloaded. The mechanical properties were calculated by using the Oliver & Pharr method and the results are shown in Fig. 14. The flowchart of the MD simulation from initial coordinates and thermal effects to obtain final trajectories of the residues chain-length of both α-1,4 and β-1,4 linkages of cellulose is displayed in Fig. 15. Donor-acceptor probabilities for H- bond formation with the oxygen functionalities into the structures with 5, 10 and 20 residues chain-length of both α-1,4 and β-1,4 linkages of cellulose is also presented in Fig. 16, Fig. 17.

## 2 Experimental design, materials, and methods

The main information on the extraction and experimental equipment used to obtain the nanocellulose samples from PWS and SBC is reported in reference [1].

The FT-IR spectra of CNCs obtained from CC, PSW and SCB were acquired in a spectrum one (Perkin Elmer) spectrometer in transmittance mode. Samples were scanned 20 times at the maximum resolution (4.0 cm^−1^) in the range of 4000-500 cm^−1^. As reference, commercial cellulose acquired from Sigma-Aldrich (type 20, 20 µm) was also analyzed.

Particle size distribution of the as-prepared CNCs samples was determinate by Dynamic Light Scattering (DLS) using a Litesizer 500 equipment (40 mW semiconductor laser of 658 nm wavelength; Particle Analyzer, Anton Paar). The nanocellulose was dispersed in pharmaceutical water (PISA), and adjusted to a concentration of 1 g L^−1^; the sample was sonicated for 20 min to achieve a good dispersion; after, it was diluted at different concentrations (0.05 mg mL^−1^).

DSC/TGA studies were realized in a LAB SYS EVO SETARAM instrumentation. 3 mg of sample was placed in a crucible. The experiments were carried out in an inert atmosphere (Ar) from 30 to 250°C in an aluminum pan with a heating and cooling rate of 5°C min^−1^ followed by cooling to 30°C to eliminate the thermal history of the materials. Then, they were heated again until 250°C.

The cellulose nanocrystalline membranes were prepared with the extracted solution of the acid hydrolysis, which was previously maintained in sonication for 2 h to minimize agglomeration. The resulting solution was then left at room temperature for 2 h for the removal of possible bubbles trapped within the solution. Thereafter, the membranes were casted with a 120 µm casting knife (film applicator, Elcometer 3580) onto a glass plate at room temperature and 80% of air humidity. The polymeric film was peeled off from the glass plate and introduced in a separate water bath for 3 h to eliminate residues. Then, the film was dried for 24 h before characterization.

The structure of the samples (membranes) was determined by X-ray diffraction (XRD) with a Bruker D8 Advance diffractometer equipped with a Lynxeye detector and Cu Kα radiation (λ = 1.5405 Å) at 40 kV and 35 mA. Data were collected at room temperature in the 2θ range of 5–50° with a step of 1° min^−1^. Data were plotted and analyzed from 10 to 40° (2θ).

The morphology of selected membranes was studied by scanning electron microscopy (SEM) using a JEOL JSM-6701F microscope (5 kV of acceleration voltage) with no conductive coating. TEM images and SAED patterns were taken in a JEOL JEM-2000 FX at 200 kV.

Mechanical properties were analyzed on rectangular membranes (25.4 × 38.1 mm) using an Anton Paar Nanoindentation Tester NHT^3^ with the Oliver & Pharr method. A Poisson's ratio of 0.38 was used for the evaluation [2]. A loading of 8 mN min^−1^ was applied until reaching a maximum load of 40 mN. The load was held for 10 s in order to account the creep effects and then the indenter was unloaded. Five measurements were acquired to obtain representative values.

Molecular dynamic (MD) simulations were performed to model the formation of cellulose type I or II based on of the number of glucosyl residues and reaction temperature. The simulated system consisted of micro-fibrils of 5, 10 and 20 glucosyl residues as a measurement of the cellulose polymerization degree (C_6_H_10_O_5_)_n_. Carbon atoms are in six-membered ring of each glucose group, being linked each other by common oxygen (O) atom (ester bond), glucose groups are polymerized into a large polymer where each molecule is interacting mainly by hydrogen bonding. Model fibrils were subjected to energy minimization (250,000 steps of minimization; 500 of steepest descent), followed by heating at 60, 75 and 90°C. All primary structures were produced using the GLYCAM carbohydrate builder based on the coordinates for cellulose 1,4-alpha and beta, prior AMBER (Assisted Model building and Energy Refinement) minimization. All simulations were carried out with SHAKE algorithm using a 2 fs time step and Langevin dynamics for temperature control [3] and for simplicity, water was used as simulated medium. The systems were stabilized by the transferable intermolecular potential 3P (TIP3P).The total potential energy of cellulose is partitioned into several energy terms. These contributions include non-bonding energies, bond stretching energies, angle bending energies, torsion angle energies, out-of-plant bending energies and coulombic interaction energies.

## References

[1] Macías-Almazán A., Lois-Correa J.A., Domínguez-Crespo M.A., López-Oyama A.B., Torres-Huerta A.M., Brachetti-Sibaja S.B., Rodríguez-Salazar A.E. (2020). Influence of operating conditions on Proton Conductivity of Nanocellulose films using two Agroindustrial Wastes: Sugarcane Bagasse and Pinewood Sawdust. Carbohydrate Polymers.

[2] Nakamura K., Wada M., Kuga S., Okano T. (2004). Poisson's of Cellulose Iα and cellulose II. Journal of Polymer Science, Part B: Polymer Physics.

[3] Adcock S.A., McCammon J.A. (2006). Molecular dynamics: survey of methods for simulating the activity of proteins. Chemical reviews.

